# Literature in Medicine

**Published:** 1864-05

**Authors:** 


Literature in Medicine.—The following from the Am. Med. Times, is applicable to the dental as well as the medical profession, so that we could not refrain from publishing it. We wish that every member of our profession would read, and consider.

"It is a noticeable fact that the greatest physicians have always been the best writers among physicians."—Zimmermann.

There is an anecdote current that a New York physician, recently traveling abroad, met a distinguished Parisian surgeon, to whom he spoke in somewhat laudatory terms of his preceptor, a well known American surgeon. "What has he done?" was the prompt inquiry of the foreigner, adding, "I

don't remember to have read any of his writings." "It is true, he has never written any thing," replied the puzzled American, "but then he has a very large business." "And is that the standard by which you estimate professional excellence?" retorted the surgeon, with a look and gesture expressive of contempt.

There is in this incident a world of meaning. It sets forth vividly a national trait in our profession, which disgraces us individually as a body. We are proud of being called practical, having no time to write, on account of the severe pressure of our business engagements. The young man who, after being located half a dozen years in practice, still goes on foot, is set down as a failure. There is no hope of his ever rising to a level with the aristocracy of his profession. It matters little what may be his scientific attainments or his moral worth; he is an object of pity, if not of contempt. Men in high and influential positions frequently boast of their incomes, and exhibit their list of daily calls, or their bank-books, as an evidence of success. Half of the gossip in professional circles relates to the income of individuals.

These false ideas of professional success have taken deep root among us, and are bearing bitter fruits. The recent graduate is driven to seek business as the first great desideratum. He abandons the pursuit of special studies, for which he may have a predilection, because they will not immediately "pay." He cannot afford to labor patiently in the pursuit of knowledge, and let business come as its sweet reward. Like Ortugal, he demands that "the golden stream be quick and violent." If patients do not immediately seek him, he goes out into the highways and byways, and compels them to come in. At all hazards he must have the apperance of business. Urged on by this infatuation, he assumes all the externals of success. His mode of living and his equipage, are often far beyond his income, but he lives in the hope that these glittering baubles will advance his business, and in the end reimburse his outlay. He *may* attain the summit of his ambition, and acquire the largest practice in the community; but it is not improbable that he will sadly fail. But whether he succeeds or not, he is lost to the science of his profession. He may seek positions in hospitals, schools, and societies, as collateral aids to success, but in every position he is a nonentity. His name may be trumpeted throughout the world, but no man of education will even recognize it. He dies and leaves behind him no memorial but the perishable marble. A short generation passes from the stage, and his memory is swept forever from the earth.

It is time the profession of this country set up a higher standard of merit than that now so generally adopted. We should pay homage only to genuine worth. The palm of excellence should be given to him who has the profoundest practical knowledge of the science and art of medicine, and who makes that knowledge available to others.

As a profession we should not only cultivate science, but we should also cultivate literary taste. History and observation prove the truth of Zimmermann's remark—that the greatest medical writers of any age were the best physicians. We have no right to ridicule the man who frequently communicates his views to the profession. While it is true that too many write who have nothing worthy of publication, it is sadly true that many who fill high places, withhold altogether their experience from their brethren. There are in this and other communities too many of this latter class. They are intellectually and morally worthy of the confidence of the profession, and capable of being the leaders in the department of practice to which they are especially devoted. By virtue of true merits, they have obtained responsible positions in our hospitals, schools, and associations, and are qualified by long experience and sound judgment to instruct. It is to them that the profession look for sound instruction in practical medicine, surgery, and obstetrics, and for a just estimate of the value of the more recent improvements. But they are sealed books that give no information. They are quick to turn every advantage which official position may have given them to their personal and pecuniary account, but they make no return to those who have raised them to power. They will have their reward in that utter oblivion which is hereafter to cover their names.

